# Microbial Coaggregation in the Oral Cavity: Molecular Interactions and Current Insights

**DOI:** 10.3390/ijms262110552

**Published:** 2025-10-30

**Authors:** Yuichi Oogai, Yumika Tanaka, Masanobu Nakata

**Affiliations:** 1 Department of Oral Microbiology, Graduate School of Medical and Dental Sciences, Kagoshima University, 8-35-1 Sakuragaoka, Kagoshima 890-8544, Japan; 2 Department of Periodontology, Graduate School of Medical and Dental Sciences, Kagoshima University, 8-35-1 Sakuragaoka, Kagoshima 890-8544, Japan

**Keywords:** periodontal pathogen, coaggregation, *Fusobacterium nucleatum*, red complex

## Abstract

Periodontitis is a chronic inflammatory disease of the periodontal tissues primarily caused by dysbiotic bacterial communities. Accumulating evidence suggests that periodontal pathogens not only drive the initiation and progression of periodontitis but also significantly contribute to systemic disorders, including diabetes mellitus, cardiovascular disease, cancer, and adverse pregnancy outcomes, such as preterm birth. The key periodontal pathogens implicated in disease pathogenesis include *Porphyromonas gingivalis*, *Prevotella intermedia*, *Treponema denticola*, *Tannerella forsythia*, *Aggregatibacter actinomycetemcomitans*, and *Fusobacterium nucleatum*. Among the diverse factors governing bacterial colonization and biofilm formation, interspecies interactions, particularly coaggregation, play a critical role in dental plaque maturation and the establishment of pathogenic microbial communities. Coaggregation facilitates the spatial organization of bacteria within biofilms, enhances bacterial survival, and modulates virulence factor expression. This review summarizes current knowledge regarding bacterial interactions involving major periodontal pathogens, with particular emphasis on coaggregation mechanisms, and discusses the implications of this coaggregation for periodontitis pathogenesis and associated systemic diseases.

## 1. Introduction

The oral cavity harbors one of the most diverse microbial ecosystems in the human body, comparable to that of the gastrointestinal tract. To date, more than 700 microbial species have been identified in the oral environment, of which approximately 500 are bacterial species [1,2]. Oral bacteria adapt to distinct ecological niches, adhering to gingival tissues and tooth surfaces, and thereby facilitating dental plaque formation. Periodontal pathogens are predominantly obligate anaerobes that colonize the subgingival crevice, where oxygen tension is relatively low. In contrast, streptococci, the dominant bacterial group in the oral cavity, exhibit greater tolerance to oxidative stress. Their early colonization of tooth surfaces facilitates the subsequent establishment of late colonizers, including periodontal pathogens [3,4]. Early and late colonizers interact with each other, either directly or indirectly, through mechanisms such as coaggregation [5,6]. Such interspecies interactions are fundamental to plaque maturation and provide a foundation for understanding how key periodontal pathogens establish themselves within the oral microbiome. Moreover, when the ecological balance of these microbial communities is disturbed by factors, such as poor oral hygiene and host immune dysregulation, the resulting dysbiosis promotes the overgrowth of pathogenic species and triggers the chronic inflammation characteristic of periodontitis [7,8]. Understanding these dysbiotic shifts is therefore crucial for elucidating the etiopathogenesis of periodontal disease. Importantly, accumulating evidence suggests that oral dysbiosis not only contributes to periodontal tissue destruction but also influences systemic health, linking periodontitis to several diseases, including cardiovascular disease, Alzheimer’s disease, diabetes, and cancer [9,10].

Several bacterial species have been recognized as major periodontal pathogens. These include *Porphyromonas gingivalis*, *Prevotella intermedia*, *Treponema denticola*, *Tannerella forsythia*, *Aggregatibacter actinomycetemcomitans*, and *Fusobacterium nucleatum*. Each of these organisms exhibits distinct virulence strategies that facilitate colonization of the periodontal niche, disruption and evasion of host defenses, and induction and perpetuation of chronic inflammation [11,12]. In the following sections, we outline the biological characteristics and pathogenic roles of these representative species, beginning with *P. gingivalis*, which has long been regarded as a keystone pathogen in periodontitis.

## 2. *Porphyromonas gingivalis*

*P. gingivalis* is a Gram-negative, obligate anaerobic, rod-shaped bacterium that derives metabolic energy primarily from protein degradation products, heme, and vitamin K. It is an opportunistic pathogen that colonizes the oral cavity and is prevalent in the human population. As a major etiological agent of chronic periodontitis, *P. gingivalis* has been extensively studied [13]. Beyond its role in periodontal disease, this bacterium has also been implicated in a variety of systemic disorders, including cardiovascular disease [14,15], Alzheimer’s disease [16,17], and rheumatoid arthritis [18,19].

Among the various virulence factors of *P. gingivalis*, gingipains represent the most prominent and extensively studied components. Gingipains comprise two types of cysteine proteases: arginine-specific gingipains (RgpA and RgpB) and lysine-specific protease (Kgp) [20,21]. RgpA and Kgp are multidomain proteins consisting of an N-terminal catalytic protease domain and multiple C-terminal adhesin (hemagglutinin/adhesin) domains, whereas RgpB contains only the catalytic domain [22]. The catalytic domains of gingipains cleave a broad range of host extracellular matrix proteins, including fibronectin, fibrinogen, laminin, type I and IV collagen, and junctional adhesion molecule-1 [23,24,25]. The adhesin domains promote attachment to host tissues and acquisition of heme and other nutrients from host proteins [26,27]. Collectively, gingipains contribute to tissue destruction and nutrient acquisition, thereby enhancing bacterial survival within the periodontal niche and contributing significantly to the pathogenesis of periodontitis.

In addition to gingipains, fimbriae represent another major virulence factor of *P. gingivalis*. Fimbriae mediate critical interactions between the bacterium and host tissues, facilitating adherence to and invasion of target sites. These filamentous structures can bind a wide range of host components, including salivary proteins, and various host cells, such as macrophages, epithelial cells, and fibroblasts [28,29,30,31,32]. Through these interactions, fimbriae contribute not only to colonization of the periodontal niche but also to modulation of host immune responses, thereby promoting bacterial persistence and disease progression.

Besides gingipains and fimbriae, *P. gingivalis* produces several other virulence factors that function synergistically to promote colonization and immune modulation. These include capsule polysaccharides that protect against phagocytosis [33], lipopolysaccharide (LPS) with atypical lipid A structures capable of modulating host inflammatory signaling [34], and outer membrane vesicles that serve as delivery vehicles for virulence factors into host tissues [35,36].

## 3. *Prevotella intermedia*

*P. intermedia* is a Gram-negative, obligate anaerobic, rod-shaped bacterium that primarily utilizes peptides, heme, and vitamin K as nutrient sources. Along with *P. gingivalis*, this organism is classified as a black-pigmented bacterium, reflecting its ability to accumulate hemin-derived pigments on blood agar plates. *P. intermedia* is commonly found in the oral cavity and is frequently detected in periodontal pockets in individuals with periodontitis [37]. As an opportunistic pathogen, it has been recognized as a significant contributor to the initiation and progression of periodontal disease [38,39]. In addition to its established role in periodontitis, this bacterium has been linked to various systemic conditions, including respiratory infections [40,41], preterm birth [42], and other adverse pregnancy outcomes [43].

*P. intermedia* produces a range of virulence factors, including fimbriae, LPS, and elastase, but proteases are considered particularly important for pathogenesis [44]. These proteolytic enzymes mediate the degradation of host immune components, such as immunoglobulins, CD14, and LPS-binding protein, which may result in enhanced survival of *P. intermedia* and increased virulence of Gram-negative bacterial species [45,46]. In addition, the enzymatic activity of dipeptidyl peptidase IV is enhanced in the presence of estradiol [47], which can serve as an alternative nutrient source to vitamin K, suggesting a potential link between *P. intermedia* and estrogen metabolism. Additionally, *P. intermedia* has been reported to suppress neutrophil function and modulate host cytokine responses, underscoring its involvement in immune evasion and the perpetuation of chronic inflammation [48,49,50].

## 4. *Treponema denticola*

*T. denticola* is a motile, Gram-negative, obligate anaerobic spirochete that is frequently detected in subgingival plaque and is strongly linked to advanced periodontal lesions, including necrotizing periodontal diseases [51,52]. Through its characteristic motility and diverse virulence mechanisms, *T. denticola* contributes to periodontal tissue destruction and chronic inflammation [53,54]. Beyond its role in periodontal disease, *T. denticola* has also been implicated in systemic conditions, including cardiovascular disorders, likely through its ability to induce inflammatory responses in host tissues [55].

Among the various virulence factors of *T. denticola*, the surface-expressed protease dentilisin represents the most extensively characterized component. Dentilisin is a trypsin-like serine protease that degrades various host proteins, such as fibronectin, laminin, and fibrinogen, thereby facilitating bacterial colonization and modulating hemostasis in periodontal tissues [56,57]. In addition to dentilisin, the major sheath protein (Msp) forms filamentous outer membrane structures and exhibits pore-forming activity against epithelial cells, as well as hemolytic and hemagglutinating activities. Moreover, Msp forms a hetero-oligomeric complex with dentilisin, which may enhance bacterial colonization [58,59,60]. The characteristic motility of *T. denticola*, driven by periplasmic flagella, further facilitates both penetration into gingival tissues and movement within the subgingival environment [61]. Collectively, these virulence factors facilitate tissue destruction in periodontal lesions.

## 5. *Tannerella forsythia*

*T. forsythia* is a Gram-negative, obligate anaerobic, rod-shaped bacterium that colonizes subgingival plaque, particularly among periodontitis patients [62]. Along with *P. gingivalis* and *T. denticola*, this organism is classified as part of the “Red complex”, a group of bacteria strongly associated with chronic periodontitis progression [63]. *T. forsythia* possesses multiple virulence factors [64], and the S-layer, a glycosylated crystalline surface layer, represents the most distinctive feature among them. The S-layer mediates complement resistance, modulates immune recognition, and contributes to multispecies biofilm formation, thereby facilitating bacterial persistence in the periodontal niche [65,66,67]. Although primarily an oral pathogen, *T. forsythia* may contribute to systemic inflammation, and its involvement in cardiovascular disease and metabolic disorders has been suggested in recent studies [68].

*T. forsythia* produces several distinct proteases, including karilysin, a matrix metalloprotease-like enzyme that degrades elastin, fibrinogen, and fibronectin [69], and mirolase, a subtilisin-like serine protease that degrades fibrinogen, hemoglobin, and the antimicrobial peptide LL-37 [70]. These proteases contribute to tissue destruction and immune evasion [69,70,71]. The bacterium also expresses BspA, an outer membrane leucine-rich repeat protein that mediates adhesion to epithelial cells and extracellular matrix components while triggering host inflammatory responses [72,73]. Furthermore, its LPS possesses immunomodulatory properties that contribute to bacterial survival and chronic inflammation.

## 6. *Aggregatibacter actinomycetemcomitans*

*A. actinomycetemcomitans* is a Gram-negative, facultative anaerobe implicated in periodontitis, particularly aggressive forms of the disease [74,75]. In addition to its role in periodontal disease, this organism has been implicated in various systemic disorders, including endocarditis [76], Alzheimer’s disease [77], and brain abscess [78].

Its major virulence factors include LPS [79], pili [80,81], leukotoxin [82], cytolethal distending toxin (CDT) [83], outer membrane proteins, such as Omp100 (ApiA) [84,85] and EmaA [86,87], and outer membrane vesicles [88]. LPS contributes to the modulation of host immune responses and induction of inflammatory signaling. Pili mediate adhesion to host cells and extracellular matrix components, facilitating colonization of the periodontal niche. Leukotoxin selectively targets polymorphonuclear leukocytes, lymphocytes, and monocytes/macrophages, impairing host immune defenses and promoting bacterial persistence. CDT induces cell cycle arrest and apoptosis in host cells, further disrupting tissue homeostasis and promoting periodontal tissue destruction. Omp100 (ApiA) mediates adhesion to epithelial cells and extracellular matrix proteins, while EmaA facilitates collagen binding and enhances biofilm formation.

## 7. *Fusobacterium nucleatum*

*F. nucleatum* is a Gram-negative, obligate anaerobic, fusiform bacterium commonly found in the human oral cavity. This organism contributes to the progression of periodontitis through multiple mechanisms, including adherence to and invasion of gingival epithelial cells [89], thereby triggering inflammatory cytokine production [90]. While its direct contribution to periodontitis is less clearly defined compared to the aforementioned periodontal pathogens, *F. nucleatum* plays a crucial role in facilitating coaggregation between early and late colonizing bacteria and promoting biofilm maturation [91,92]. Beyond its oral pathogenic potential, *F. nucleatum* has also been implicated in systemic diseases, including atherosclerotic cardiovascular disease, adverse pregnancy outcomes, inflammatory bowel disease, and cancer, with particular attention given to its strong association with colorectal cancer, which has been extensively investigated in recent years [93,94].

Key virulence factors of *F. nucleatum* include the cell surface adhesin FadA and the outer membrane autotransporter protein Fap2, which mediate both interbacterial interactions and adhesion to host cells. FadA binds to E-cadherin on epithelial cells, facilitating bacterial invasion and activation of β-catenin signaling, which promotes pro-inflammatory responses and may contribute to colorectal tumorigenesis [95,96]. Fap2 interacts with host immune cells and other bacteria, enhancing coaggregation and biofilm maturation while inhibiting natural killer cell activity, thereby modulating host immune defenses [97,98,99,100]. Through these virulence factors, *F. nucleatum* functions as a bridging organism in dental plaque, promotes persistent inflammation in the oral cavity, and supports colonization and progression of colorectal cancer, illustrating its dual role in both local and systemic pathogenesis.

## 8. Coaggregation Between *F. nucleatum* and Oral-Bacterial Species

*F. nucleatum* actively mediates the bridging of early and late colonizers within the oral cavity by coaggregating with a wide range of bacterial species, thereby facilitating dental plaque maturation [101]. In this section, we highlight representative examples of oral-bacterial species that coaggregate with *F. nucleatum* and summarize the molecular determinants underlying these interactions. Table 1 summarizes oral bacterial species that coaggregate with *F. nucleatum*, along with the molecules involved in these interactions and the molecules that inhibit them.

**Figure 1:**
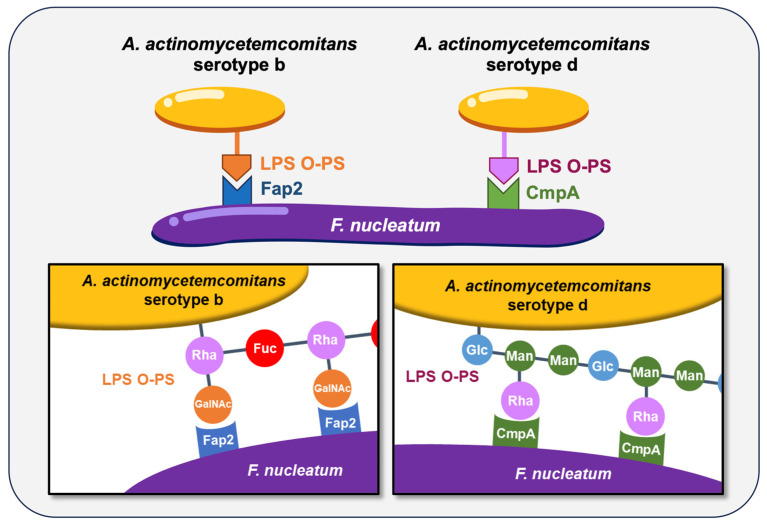
The coaggregation of *F. nucleatum* with *A. actinomycetemcomitans* serotypes b and d. These coaggregations are mediated by specific surface proteins and O-polysaccharide (O-PS) regions of lipopolysaccharide (LPS). Rha, _L_-rhamnose; Fuc, _D_-fucose; GalNAc, *N*-acetyl-_D_-galactosamine; Glc, _D_-glucose; Man, _D_-mannose.

The coaggregation between *F. nucleatum* and *A. actinomycetemcomitans* has long been characterized, and this interaction is known to be serotype-dependent. *A. actinomycetemcomitans* is classified into seven serotypes (a, b, c, d, e, f, and g) based on the antigenicity of the O-polysaccharide (O-PS) regions of its LPS [102,103,104,105]. Strains reported to coaggregate with *F. nucleatum* include Y4 (serotype b) [91], SA269 (serotype d) [106], and CU1060N (serotype f) [107]. More recently, we demonstrated that coaggregation between *A. actinomycetemcomitans* strains HK1651 (serotype b) and IDH781 (serotype d) and *F. nucleatum* is mediated by serotype-specific recognition of O-PS, with *F. nucleatum* utilizing the autotransporter proteins Fap2 and CmpA, respectively [108] (Figure 1). These findings highlight that bacterial coaggregation can vary not only between species but also among strains within the same species, underscoring the complexity of interbacterial interactions in the oral cavity. Additionally, the autotransporter protein RadD exhibits coaggregation activity to *A. actinomycetemcomitans* JP2 (serotype b) [109].

*P. gingivalis* has been shown to coaggregate with *F. nucleatum*. The coaggregation between *F. nucleatum* PK1594 and *P. gingivalis* PK1924 has been reported to be inhibited by lactose, *N*-acetyl-_D_-galactosamine, and _D_-galactose [110,111]. Galactose-dependent coaggregation was also observed in *F. nucleatum* ATCC 23726, and screening of mutants defective in this phenotype identified Fap2 as a coaggregation factor of *F. nucleatum* [109,112]. Additionally, deletion of the porin protein FomA in *P. gingivalis* ATCC 33277 reduced its coaggregation with *F. nucleatum* ATCC 10953 [113].

Coaggregation between *F. nucleatum* ATCC 25586 and *P. intermedia* ATCC 25611 is inhibited by EDTA and *N*-acetyl-_D_-galactosamine [114]. These findings suggest a coaggregation mechanism similar to that observed with *P. gingivalis*, although the specific coaggregation factors have not yet been identified. This interaction was suppressed by heat or protease treatment of *F. nucleatum*, but not by the same treatments applied to *P. intermedia* [114], suggesting that the coaggregation factor on the *P. intermedia* side is likely a galactose-containing surface polysaccharide.

The molecular mechanisms underlying coaggregation between *T. forsythia* and *F. nucleatum* have not been extensively characterized. Coaggregation between *F. nucleatum* ATCC 10953 and *T. forsythia* ATCC 43037 was attenuated by deletion of the surface adhesin BspA in *T. forsythia* [115]. We also reported that *F. nucleatum* ATCC 25586 strongly coaggregates with *T. forsythia* ATCC 43037, and that coaggregation was enhanced when *T. forsythia* lacked the S-layer [65]. Since the S-layer of *T. forsythia* is glycosylated [116], inhibition assays were performed using sugars associated with S-layer glycosylation; however, these sugars did not suppress coaggregation between *F. nucleatum* and wild-type *T. forsythia* [65]. These findings suggest that surface proteins, such as BspA, rather than the S-layer itself, may be responsible for mediating the coaggregation.

*F. nucleatum* PK1594 coaggregates with *T. denticola* strains ATCC 35404, ATCC 33520, and GM-1. This coaggregation is inhibited by the addition of EDTA and galactose. Msp of *T. denticola* is modified with galactose-containing glycans, and purified Msp inhibited the binding between *F. nucleatum* and *T. denticola* in a concentration-dependent manner [117]. However, deletion of Msp did not reduce interbacterial binding, suggesting that multiple coaggregation factors may mediate this interaction in *T. denticola* [117].

In addition to periodontal pathogens, it has also been reported that *F. nucleatum* coaggregates with the oral commensals *Actinomyces oris*, *Actinomyces naeslundii*, *Streptococcus gordonii*, *Streptococcus oralis*, and *Streptococcus sanguinis* through its autotransporter proteins RadD and/or CmpA [118,119,120]. Many of these coaggregation interactions are inhibited by the addition of arginine [118]. Wu et al. performed a genome-wide screening of *F. nucleatum* to identify genes involved in coaggregation with *S. gordonii*, and identified genes within the *rad* operon, *carS* encoding a histidine kinase, and those related to the lysine degradation pathway [109]. Furthermore, they found that *radD* and genes involved in lysine metabolism are regulated by the CarRS two-component system, suggesting that lysine metabolism induced by CarRS regulation may contribute to coaggregation with *S. gordonii* [109]. Disruption of CarRS-regulated genes involved in lysine metabolism led to increased lysine concentrations in the culture supernatant [109], which suggests that RadD-mediated coaggregation may be modulated by both arginine and lysine.

**Table 1:** Coaggregation partners of *F. nucleatum*.

Partner Species	Coaggregation Factor of *F. nucleatum*	Coaggregation Factor of Partner ^1^	Inhibitor ^2^	Reference
*A. actinomycetemcomitans* HK1651	Fap2	Serotype b O-PS	GalNac, Rha	[108]
*A. actinomycetemcomitans* IDH781	CmpA	Serotype d O-PS	Rha	[108]
*A. actinomycetemcomitans* JP2	RadD	Unidentified	-	[109]
*P. gingivalis* PK1924	Unidentified	Unidentified	Lac, Gal, GalNac	[110]
*P. gingivalis* PK1924	Unidentified	CPS, LPS	EDTA, Gal	[111]
*P. gingivalis* PK1924	Fap2	Unidentified	Gal	[112]
*P. gingivalis* PK1924	Fap2	Unidentified	-	[109]
*P. gingivalis* ATCC 33277	FomA	Unidentified	-	[113]
*P. intermedia* ATCC 25611	Unidentified	Unidentified	EDTA, GalNac	[114]
*T. forsythia* ATCC 43037	Unidentified	BspA	-	[115]
*T. forsythia* ATCC 43037	Unidentified	Unidentified	-	[65]
*T. denticola* ATCC 35404, ATCC 33520, GM-1	Unidentified	Msp	EDTA, Gal	[117]
*A. oris* MG-1	RadD	Unidentified	-	[119]
*A. naeslundii* ATCC 12104	RadD	Unidentified	_L_-arginine	[118]
*S. gordonii* ATCC 10558	RadD	Unidentified	_L_-arginine	[118]
*S. gordonii* ATCC 10558, ATCC 51656,DL1	RadD, CmpA	Unidentified	-	[120]
*S. gordonii* DL1	RadD	Unidentified	-	[109]
*S. oralis* ATCC 10557	RadD	Unidentified	_L_-arginine	[118]
*S. sanguinis* ATCC 10556	RadD	Unidentified	_L_-arginine	[118]

^1^ O-PS, O-polysaccharide; CPS, capsule polysaccharide; LPS, lipopolysaccharide. ^2^ GalNac, *N*-acetyl-_D_-galactosamine; Rha, rhamnose; Lac, lactose; Gal, galactose.

## 9. Coaggregation Between Red Complex Species

In chronic periodontitis lesions, the so-called “Red complex” species—*P. gingivalis*, *T. denticola*, and *T. forsythia*—are frequently co-isolated and are considered the major periodontal pathogens. In this section, we summarize reports describing coaggregation among these bacterial species (Table 2).

Regarding the coaggregation between *P. gingivalis* and *T. denticola*, several studies have identified molecular factors involved in this interaction. Hashimoto et al. demonstrated that dentilisin of *T. denticola* can bind to fimbrial proteins of *P. gingivalis*, suggesting its potential role in coaggregation [121]. Yamada et al. reported that *P. gingivalis* strain FDC381 and *T. denticola* strain ATCC 35405 coaggregate strongly, and that this interaction is reduced when *T. denticola* mutant strains lacking *flgE* (flagellar gene) or *cfpA* (cytoplasmic filament gene) are used [122]. Furthermore, Yoshikawa et al. showed that the Hgp44 domain of RgpA in *P. gingivalis* is critically involved in coaggregation between *P. gingivalis* ATCC 33277 and *T. denticola* ATCC 35405 [123].

Concerning the coaggregation between *P. gingivalis* and *T. forsythia*, Jung et al. reported that the interaction between *P. gingivalis* ATCC 33277 and *T. forsythia* ATCC 43037 is strongly inhibited by _L_-arginine and _L_-lysine, and that a gingipain-null mutant of *P. gingivalis* (*kgp*^−^, *rgpA*^−^, *rgpB*^−^) completely lost its ability to coaggregate with *T. forsythia* [124]. Additionally, Śmiga et al. investigated the role of the redox-sensing protein (PgRsp) in *P. gingivalis* and found that a *pgRsp*-deficient mutant of strain A7436 displayed enhanced coaggregation with *T. forsythia* ATCC 43037. This phenotype was attributed to downregulated fimbrial gene expression in the *pgRsp* mutant, which may increase the exposure of other *P. gingivalis* surface factors and thereby promote coaggregation [125].

In the case of coaggregation between *T. denticola* and *T. forsythia*, Ikegami et al. demonstrated that the leucine-rich repeat protein LrrA of *T. denticola* ATCC 35405 mediates coaggregation with *T. forsythia* ATCC 43037 by interacting with the surface protein BspA of *T. forsythia* [126]. Furthermore, Sano et al. confirmed the coaggregation between *T. denticola* ATCC 35405 and *T. forsythia* ATCC 43037 and showed that the surface protease dentilisin of *T. denticola* contributes to this interaction [127].

## 10. Conclusions and Perspectives

Oral bacteria engage in a wide range of interspecies coaggregation events, with *Fusobacterium nucleatum* serving as a central bridging organism. Major periodontal pathogens coaggregate not only through *F. nucleatum* but also directly with each other, and such microbial colocalization is thought to promote the progression and exacerbation of periodontitis. Importantly, *F. nucleatum* also interacts with diverse bacterial species outside the oral cavity, including *Staphylococcus aureus* on the skin, *Helicobacter pylori* in the stomach, and *Clostridioides difficile* and *Limosilactobacillus reuteri* in the intestine [128,129,130,131]. These broad coaggregation capabilities may facilitate the dissemination of periodontal pathogens beyond the oral environment and provide a mechanistic link to systemic diseases. Future research should focus on elucidating the detailed molecular mechanisms underlying these coaggregation events and exploring their potential as therapeutic targets. Additionally, the role of coaggregation in establishing dysbiotic microbial communities warrants further investigation.

## Figures and Tables

**Table 2:** Coaggregations between Red complex species.

Coaggregation	Coaggregation Factor ^1^		Reference
*P. gingivalis*–*T. denticola*	Fimbriae	Dentilisin	[121]
	-	FlgE, CfpA	[122]
	RgpA	-	[123]
*P. gingivalis*–*T. forsythia*	RgpA, RgpB, Kgp	-	[124]
	-	-	[125]
*T. denticola*–*T. forsythia*	LrrA	BspA	[126]
	Dentilisin	-	[127]

^1^ The left column indicates the coaggregation factors of *P. gingivalis* or *T. denticola*. The right column indicates the coaggregation factors of *T. denticola* or *T. forsythia*.

## Data Availability

This review article relies exclusively on previously published data, fully cited in the bibliography. The original datasets used in the cited studies were not accessed or analyzed by the authors.

## References

[1] Aas J.A., Paster B.J., Stokes L.N., Olsen I., Dewhirst F.E. (2005). Defining the normal bacterial flora of the oral cavity. J. Clin. Microbiol..

[2] Paster B.J., Boches S.K., Galvin J.L., Ericson R.E., Lau C.N., Levanos V.A., Sahasrabudhe A., Dewhirst F.E. (2001). Bacterial diversity in human subgingival plaque. J. Bacteriol..

[3] Loesche W.J., Gusberti F., Mettraux G., Higgins T., Syed S. (1983). Relationship between oxygen tension and subgingival bacterial flora in untreated human periodontal pockets. Infect. Immun..

[4] Nyvad B., Kilian M. (1987). Microbiology of the early colonization of human enamel and root surfaces in vivo. Scand. J. Dent. Res..

[5] Bradshaw D.J., Marsh P.D., Watson G.K., Allison C. (1998). Role of *Fusobacterium nucleatum* and coaggregation in anaerobe survival in planktonic and biofilm oral microbial communities during aeration. Infect. Immun..

[6] Periasamy S., Kolenbrander P.E. (2009). Mutualistic biofilm communities develop with *Porphyromonas gingivalis* and initial, early, and late colonizers of enamel. J. Bacteriol..

[7] DeGruttola A.K., Low D., Mizoguchi A., Mizoguchi E. (2016). Current understanding of dysbiosis in disease in human and animal models. Inflamm. Bowel Dis..

[8] Chin S.W., Low Z.Y., Tan W.Q., Azman A.S. (2025). Microbiota-host interactions: Exploring their dynamics and contributions to human diseases. Microbiologyopen.

[9] Boyapati R., Vudathaneni V.K.P., Bodduru R., Todima J., Dasari A.B., Chintala L. (2025). Exploring the link between periodontal pathogens and systemic inflammatory markers in patients with metabolic syndrome. J. Pharm. Bioallied Sci..

[10] Murray P.E., Coffman J.A., Garcia-Godoy F. (2024). Oral pathogens’ substantial burden on cancer, cardiovascular diseases, Alzheimer’s, diabetes, and other systemic diseases: A public health crisis-A comprehensive review. Pathogens.

[11] Socransky S.S., Haffajee A.D. (2005). Periodontal microbial ecology. Periodontology 2000.

[12] Hajishengallis G. (2015). Periodontitis: From microbial immune subversion to systemic inflammation. Nat. Rev. Immunol..

[13] Polishchuk H., Synowiec A., Zubrzycka N., Kantyka T. (2025). *Porphyromonas gingivalis*: Multiple tools of an inflammatory damage. Mol. Oral Microbiol..

[14] Pavlic V., Peric D., Kalezic I.S., Madi M., Bhat S.G., Brkic Z., Staletovic D. (2021). Identification of periopathogens in atheromatous plaques obtained from carotid and coronary arteries. Biomed. Res. Int..

[15] Ruan Q., Guan P., Qi W., Li J., Xi M., Xiao L., Zhong S., Ma D., Ni J. (2023). *Porphyromonas gingivalis* regulates atherosclerosis through an immune pathway. Front. Immunol..

[16] Dominy S.S., Lynch C., Ermini F., Benedyk M., Marczyk A., Konradi A., Nguyen M., Haditsch U., Raha D., Griffin C. (2019). *Porphyromonas gingivalis* in Alzheimer’s disease brains: Evidence for disease causation and treatment with small-molecule inhibitors. Sci. Adv..

[17] Díaz-Zúñiga J., More J., Melgar-Rodríguez S., Jiménez-Unión M., Villalobos-Orchard F., Muñoz-Manríquez C., Monasterio G., Valdés J.L., Vernal R., Paula-Lima A. (2020). Alzheimer’s disease-like pathology triggered by *Porphyromonas gingivalis* in wild type rats is serotype dependent. Front. Immunol..

[18] Maresz K.J., Hellvard A., Sroka A., Adamowicz K., Bielecka E., Koziel J., Gawron K., Mizgalska D., Marcinska K.A., Benedyk M. (2013). *Porphyromonas gingivalis* facilitates the development and progression of destructive arthritis through its unique bacterial peptidylarginine deiminase (PAD). PLoS Pathog..

[19] Yamakawa M., Ouhara K., Kajiya M., Munenaga S., Kittaka M., Yamasaki S., Takeda K., Takeshita K., Mizuno N., Fujita T. (2016). *Porphyromonas gingivalis* infection exacerbates the onset of rheumatoid arthritis in SKG mice. Clin. Exp. Immunol..

[20] Nakayama K., Kadowaki T., Okamoto K., Yamamoto K. (1995). Construction and characterization of arginine-specific cysteine proteinase (Arg-gingipain)-deficient mutants of *Porphyromonas gingivalis*. Evidence for significant contribution of Arg-gingipain to virulence. J. Biol. Chem..

[21] Lei Z., Ma Q., Zhou X., Li Y. (2025). The secretion and maturation journey of gingipains. Mol. Oral Microbiol..

[22] Li N., Collyer C.A. (2011). Gingipains from *Porphyromonas gingivalis*—Complex domain structures confer diverse functions. Eur. J. Microbiol. Immunol..

[23] Baba A., Abe N., Kadowaki T., Nakanishi H., Ohishi M., Asao T., Yamamoto K. (2001). Arg-gingipain is responsible for the degradation of cell adhesion molecules of human gingival fibroblasts and their death induced by *Porphyromonas gingivalis*. Biol. Chem..

[24] Potempa J., Banbula A., Travis J. (2000). Role of bacterial proteinases in matrix destruction and modulation of host responses. Periodontology 2000.

[25] Takeuchi H., Sasaki N., Yamaga S., Kuboniwa M., Matsusaki M., Amano A. (2019). *Porphyromonas gingivalis* induces penetration of lipopolysaccharide and peptidoglycan through the gingival epithelium via degradation of junctional adhesion molecule 1. PLoS Pathog..

[26] Shi Y., Ratnayake D.B., Okamoto K., Abe N., Yamamoto K., Nakayama K. (1999). Genetic analyses of proteolysis, hemoglobin binding, and hemagglutination of *Porphyromonas gingivalis*. Construction of mutants with a combination of *rgpA*, *rgpB*, *kgp*, and *hagA*. J. Biol. Chem..

[27] DeCarlo A.A., Paramaesvaran M., Yun P.L., Collyer C., Hunter N. (1999). Porphyrin-mediated binding to hemoglobin by the HA2 domain of cysteine proteinases (gingipains) and hemagglutinins from the periodontal pathogen *Porphyromonas gingivalis*. J. Bacteriol..

[28] Nakamura T., Amano A., Nakagawa I., Hamada S. (1999). Specific interactions between *Porphyromonas gingivalis* fimbriae and human extracellular matrix proteins. FEMS Microbiol. Lett..

[29] Amano A., Nakamura T., Kimura S., Morisaki I., Nakagawa I., Kawabata S., Hamada S. (1999). Molecular interactions of *Porphyromonas gingivalis* fimbriae with host proteins: Kinetic analyses based on surface plasmon resonance. Infect. Immun..

[30] Yilmaz O., Watanabe K., Lamont R.J. (2002). Involvement of integrins in fimbriae-mediated binding and invasion by *Porphyromonas gingivalis*. Cell Microbiol..

[31] Takeshita A., Murakami Y., Yamashita Y., Ishida M., Fujisawa S., Kitano S., Hanazawa S. (1998). *Porphyromonas gingivalis* fimbriae use β2 integrin (CD11/CD18) on mouse peritoneal macrophages as a cellular receptor, and the CD18 β chain plays a functional role in fimbrial signaling. Infect. Immun..

[32] Nakagawa I., Amano A., Kuboniwa M., Nakamura T., Kawabata S., Hamada S. (2002). Functional differences among FimA variants of *Porphyromonas gingivalis* and their effects on adhesion to and invasion of human epithelial cells. Infect. Immun..

[33] Singh A., Wyant T., Anaya-Bergman C., Aduse-Opoku J., Brunner J., Laine M.L., Curtis M.A., Lewis J.P. (2011). The capsule of *Porphyromonas gingivalis* leads to a reduction in the host inflammatory response, evasion of phagocytosis, and increase in virulence. Infect. Immun..

[34] Darveau R.P., Pham T.T., Lemley K., Reife R.A., Bainbridge B.W., Coats S.R., Howald W.N., Way S.S., Hajjar A.M. (2004). *Porphyromonas gingivalis* lipopolysaccharide contains multiple lipid A species that functionally interact with both Toll-like receptors 2 and 4. Infect. Immun..

[35] Furuta N., Takeuchi H., Amano A. (2009). Entry of *Porphyromonas gingivalis* outer membrane vesicles into epithelial cells causes cellular functional impairment. Infect. Immun..

[36] Wu Z., Long W., Yin Y., Tan B., Liu C., Li H., Ge S. (2025). Outer membrane vesicles of *Porphyromonas gingivalis*: Recent advances in pathogenicity and associated mechanisms. Front. Microbiol..

[37] Ashimoto A., Chen C., Bakker I., Slots J. (1996). Polymerase chain reaction detection of 8 putative periodontal pathogens in subgingival plaque of gingivitis and advanced periodontitis lesions. Oral Microbiol. Immunol..

[38] Yang N.Y., Zhang Q., Li J.L., Yang S.H., Shi Q. (2014). Progression of periodontal inflammation in adolescents is associated with increased number of *Porphyromonas gingivalis*, *Prevotella intermedia*, *Tannerella forsythensis*, and *Fusobacterium nucleatum*. Int. J. Paediatr. Dent..

[39] Maeda N., Okamoto M., Kondo K., Ishikawa H., Osada R., Tsurumoto A., Fujita H. (1998). Incidence of *Prevotella intermedia* and *Prevotella nigrescens* in periodontal health and disease. Microbiol. Immunol..

[40] Nagaoka K., Yanagihara K., Morinaga Y., Nakamura S., Harada T., Hasegawa H., Izumikawa K., Ishimatsu Y., Kakeya H., Nishimura M. (2014). *Prevotella intermedia* induces severe bacteremic pneumococcal pneumonia in mice with upregulated platelet-activating factor receptor expression. Infect. Immun..

[41] Ashizawa H., Iwanaga N., Nemoto K., Hirayama T., Yoshida M., Takeda K., Ide S., Tashiro M., Hosogaya N., Takazono T. (2025). *Prevotella intermedia* synergistically exacerbates pneumonia induced by oral streptococci. J. Infect. Dis..

[42] Jang H., Patoine A., Wu T.T., Castillo D.A., Xiao J. (2021). Oral microflora and pregnancy: A systematic review and meta-analysis. Sci. Rep..

[43] Shira Davenport E. (2010). Preterm low birthweight and the role of oral bacteria. J. Oral. Microbiol..

[44] Zhang S., Zhao Y., Lalsiamthara J., Peng Y., Qi L., Deng S., Wang Q. (2025). Current research progress on *Prevotella intermedia* and associated diseases. Crit. Rev. Microbiol..

[45] Deschner J., Singhal A., Long P., Liu C.C., Piesco N., Agarwal S. (2003). Cleavage of CD14 and LBP by a protease from *Prevotella intermedia*. Arch. Microbiol..

[46] Jansen H.J., Grenier D., Van der Hoeven J.S. (1995). Characterization of immunoglobulin G-degrading proteases of *Prevotella intermedia* and *Prevotella nigrescens*. Oral. Microbiol. Immunol..

[47] Fteita D., Könönen E., Gürsoy M., Söderling E., Gürsoy U.K. (2015). Does estradiol have an impact on the dipeptidyl peptidase IV enzyme activity of the *Prevotella intermedia* group bacteria?. Anaerobe.

[48] Doke M., Fukamachi H., Morisaki H., Arimoto T., Kataoka H., Kuwata H. (2017). Nucleases from *Prevotella intermedia* can degrade neutrophil extracellular traps. Mol. Oral Microbiol..

[49] Sugawara S., Yang S., Iki K., Hatakeyama J., Tamai R., Takeuchi O., Akashi S., Espevik T., Akira S., Takada H. (2001). Monocytic cell activation by nonendotoxic glycoprotein from *Prevotella intermedia* ATCC 25611 is mediated by Toll-like receptor 2. Infect Immun.

[50] Guan S.M., Shu L., Fu S.M., Liu B., Xu X.L., Wu J.Z. (2008). *Prevotella intermedia* induces matrix metalloproteinase-9 expression in human periodontal ligament cells. FEMS Microbiol. Lett..

[51] Asai Y., Jinno T., Igarashi H., Ohyama Y., Ogawa T. (2002). Detection and quantification of oral treponemes in subgingival plaque by real-time PCR. J. Clin. Microbiol..

[52] Sela M.N. (2001). Role of *Treponema denticola* in periodontal diseases. Crit. Rev. Oral Biol. Med..

[53] Rosen G., Sela M.N., Naor R., Halabi A., Barak V., Shapira L. (1999). Activation of murine macrophages by lipoprotein and lipooligosaccharide of *Treponema denticola*. Infect. Immun..

[54] Malone E.T., Ganther S., Mena N., Radaic A., Shariati K., Kindberg A., Tafolla C., Kamarajan P., Fenno J.C., Zhan L. (2021). *Treponema denticola*-Induced RASA4 upregulation mediates cytoskeletal dysfunction and MMP-2 activity in periodontal fibroblasts. Front. Cell Infect. Microbiol..

[55] Okuda T., Kimizuka R., Miyamoto M., Kato T., Yamada S., Okuda K., Ishihara K. (2007). *Treponema denticola* induces interleukin-8 and macrophage chemoattractant protein 1 production in human umbilical vein epithelial cells. Microbes Infect..

[56] Uitto V.J., Pan Y.M., Leung W.K., Larjava H., Ellen R.P., Finlay B.B., McBride B.C. (1995). Cytopathic effects of *Treponema denticola* chymotrypsin-like proteinase on migrating and stratified epithelial cells. Infect. Immun..

[57] Bamford C.V., Fenno J.C., Jenkinson H.F., Dymock D. (2007). The chymotrypsin-like protease complex of *Treponema denticola* ATCC 35405 mediates fibrinogen adherence and degradation. Infect. Immun..

[58] Egli C., Leung W.K., Müller K.H., Hancock R.E., McBride B.C. (1993). Pore-forming properties of the major 53-kilodalton surface antigen from the outer sheath of *Treponema denticola*. Infect. Immun..

[59] Fenno J.C., Hannam P.M., Leung W.K., Tamura M., Uitto V.J., McBride B.C. (1998). Cytopathic effects of the major surface protein and the chymotrypsinlike protease of *Treponema denticola*. Infect. Immun..

[60] Zhao Y., Chen J., Tian Y., Huang H., Zhao F., Deng X. (2025). *Treponema denticola* major surface protein (Msp): A key player in periodontal pathogenicity and immune evasion. Arch. Microbiol..

[61] Ruby J.D., Li H., Kuramitsu H., Norris S.J., Goldstein S.F., Buttle K.F., Charon N.W. (1997). Relationship of *Treponema denticola* periplasmic flagella to irregular cell morphology. J. Bacteriol..

[62] Tanner A.C.R., Listgarten M.A., Ebersole J.L., Strzempko M.N. (1986). *Bacteroides forsythus* sp. nov. a slow-growing, fusiform *Bacteroides* sp. from the human oral cavity. Int. J. Syst. Evol. Microbiol..

[63] Mohanty R., Asopa S.J., Joseph M.D., Singh B., Rajguru J.P., Saidath K., Sharma U. (2019). Red complex: Polymicrobial conglomerate in oral flora: A review. J. Family Med. Prim. Care.

[64] Schäffer C., Andrukhov O. (2024). The intriguing strategies of *Tannerella forsythia*’s host interaction. Front. Oral Health.

[65] Shimotahira N., Oogai Y., Kawada-Matsuo M., Yamada S., Fukutsuji K., Nagano K., Yoshimura F., Noguchi K., Komatsuzawa H. (2013). The surface layer of *Tannerella forsythia* contributes to serum resistance and oral bacterial coaggregation. Infect. Immun..

[66] Settem R.P., Honma K., Nakajima T., Phansopa C., Roy S., Stafford G.P., Sharma A. (2013). A bacterial glycan core linked to surface (S)-layer proteins modulates host immunity through Th17 suppression. Mucosal Immunol..

[67] Bloch S., Thurnheer T., Murakami Y., Belibasakis G.N., Schäffer C. (2017). Behavior of two *Tannerella forsythia* strains and their cell surface mutants in multispecies oral biofilms. Mol. Oral Microbiol..

[68] Lee H.R., Jun H.K., Choi B.K. (2014). *Tannerella forsythia* BspA increases the risk factors for atherosclerosis in ApoE^−/−^ mice. Oral Dis..

[69] Karim A.Y., Kulczycka M., Kantyka T., Dubin G., Jabaiah A., Daugherty P.S., Thogersen I.B., Enghild J.J., Nguyen K.A., Potempa J. (2010). A novel matrix metalloprotease-like enzyme (karilysin) of the periodontal pathogen *Tannerella forsythia* ATCC 43037. Biol. Chem..

[70] Ksiazek M., Karim A.Y., Bryzek D., Enghild J.J., Thøgersen I.B., Koziel J., Potempa J. (2015). Mirolase, a novel subtilisin-like serine protease from the periodontopathogen *Tannerella forsythia*. Biol. Chem..

[71] Jusko M., Potempa J., Karim A.Y., Ksiazek M., Riesbeck K., Garred P., Eick S., Blom A.M. (2012). A metalloproteinase karilysin present in the majority of *Tannerella forsythia* isolates inhibits all pathways of the complement system. J. Immunol..

[72] Sharma A., Sojar H.T., Glurich I., Honma K., Kuramitsu H.K., Genco R.J. (1998). Cloning, expression, and sequencing of a cell surface antigen containing a leucine-rich repeat motif from *Bacteroides forsythus* ATCC 43037. Infect. Immun..

[73] Inagaki S., Onishi S., Kuramitsu H.K., Sharma A. (2006). *Porphyromonas gingivalis* vesicles enhance attachment, and the leucine-rich repeat BspA protein is required for invasion of epithelial cells by “*Tannerella forsythia*”. Infect. Immun..

[74] Fine D.H., Patil A.G., Velusamy S.K. (2019). *Aggregatibacter actinomycetemcomitans* (Aa) under the radar: Myths and misunderstandings of Aa and its role in aggressive periodontitis. Front. Immunol..

[75] Talapko J., Juzbašić M., Meštrović T., Matijević T., Mesarić D., Katalinić D., Erić S., Milostić-Srb A., Flam J., Škrlec I. (2024). *Aggregatibacter actinomycetemcomitans*: From the oral cavity to the heart valves. Microorganisms.

[76] Nakano K., Inaba H., Nomura R., Nemoto H., Tamura K., Miyamoto E., Yoshioka H., Taniguchi K., Amano A., Ooshima T. (2007). Detection and serotype distribution of *Actinobacillus actinomycetemcomitans* in cardiovascular specimens from Japanese patients. Oral Microbiol. Immunol..

[77] Díaz-Zúñiga J., Muñoz Y., Melgar-Rodríguez S., More J., Bruna B., Lobos P., Monasterio G., Vernal R., Paula-Lima A. (2019). Serotype b of *Aggregatibacter actinomycetemcomitans* triggers pro-inflammatory responses and amyloid beta secretion in hippocampal cells: A novel link between periodontitis and Alzheimer’s disease?. J. Oral Microbiol..

[78] Zijlstra E.E., Swart G.R., Godfroy F.J., Degener J.E. (1992). Pericarditis, pneumonia and brain abscess due to a combined *Actinomyces*—*Actinobacillus actinomycetemcomitans* infection. J. Infect..

[79] Ahlstrand T., Kovesjoki L., Maula T., Oscarsson J., Ihalin R. (2019). *Aggregatibacter actinomycetemcomitans* LPS binds human interleukin-8. J. Oral Microbiol..

[80] Kachlany S.C., Planet P.J., Desalle R., Fine D.H., Figurski D.H., Kaplan J.B. (2001). *flp-1*, the first representative of a new pilin gene subfamily, is required for non-specific adherence of *Actinobacillus actinomycetemcomitans*. Mol. Microbiol..

[81] Bhattacharjee M.K., Kachlany S.C., Fine D.H., Figurski D.H. (2001). Nonspecific adherence and fibril biogenesis by *Actinobacillus actinomycetemcomitans*: TadA protein is an ATPase. J. Bacteriol..

[82] Johansson A. (2011). *Aggregatibacter actinomycetemcomitans* leukotoxin: A powerful tool with capacity to cause imbalance in the host inflammatory response. Toxins.

[83] Sugai M., Kawamoto T., Pérès S.Y., Ueno Y., Komatsuzawa H., Fujiwara T., Kurihara H., Suginaka H., Oswald E. (1998). The cell cycle-specific growth-inhibitory factor produced by *Actinobacillus actinomycetemcomitans* is a cytolethal distending toxin. Infect. Immun..

[84] Asakawa R., Komatsuzawa H., Kawai T., Yamada S., Goncalves R.B., Izumi S., Fujiwara T., Nakano Y., Suzuki N., Uchida Y. (2003). Outer membrane protein 100, a versatile virulence factor of *Actinobacillus actinomycetemcomitans*. Mol. Microbiol..

[85] Yue G., Kaplan J.B., Furgang D., Mansfield K.G., Fine D.H. (2007). A second *Aggregatibacter actinomycetemcomitans* autotransporter adhesin exhibits specificity for buccal epithelial cells in humans and old world primates. Infect. Immun..

[86] Mintz K.P. (2004). Identification of an extracellular matrix protein adhesin, EmaA, which mediates the adhesion of *Actinobacillus actinomycetemcomitans* to collagen. Microbiology.

[87] Ruiz T., Lenox C., Radermacher M., Mintz K.P. (2006). Novel surface structures are associated with the adhesion of *Actinobacillus actinomycetemcomitans* to collagen. Infect. Immun..

[88] Fu Y., Trautwein-Schult A., Piersma S., Sun C., Westra J., de Jong A., Becher D., van Dijl J.M. (2025). Characterization of outer membrane vesicles of *Aggregatibacter actinomycetemcomitans* serotypes a, b and c and their interactions with human neutrophils. Int. J. Med. Microbiol..

[89] Han Y.W., Shi W., Huang G.T., Kinder Haake S., Park N.H., Kuramitsu H., Genco R.J. (2000). Interactions between periodontal bacteria and human oral epithelial cells: *Fusobacterium nucleatum* adheres to and invades epithelial cells. Infect. Immun..

[90] Han Y.W. (2015). *Fusobacterium nucleatum*: A commensal-turned pathogen. Curr. Opin. Microbiol..

[91] Kolenbrander P.E., Andersen R.N., Moore L.V. (1989). Coaggregation of *Fusobacterium nucleatum*, *Selenomonas flueggei*, *Selenomonas infelix*, *Selenomonas noxia*, and *Selenomonas sputigena* with strains from 11 genera of oral bacteria. Infect. Immun..

[92] Guo L., He X., Shi W. (2014). Intercellular communications in multispecies oral microbial communities. Front. Microbiol..

[93] Jiang S.S., Chen Y.X., Fang J.Y. (2025). *Fusobacterium nucleatum*: Ecology, pathogenesis and clinical implications. Nat. Rev. Microbiol..

[94] Fan Z., Tang P., Li C., Yang Q., Xu Y., Su C., Li L. (2023). *Fusobacterium nucleatum* and its associated systemic diseases: Epidemiologic studies and possible mechanisms. J. Oral Microbiol..

[95] Ikegami A., Chung P., Han Y.W. (2009). Complementation of the *fadA* mutation in *Fusobacterium nucleatum* demonstrates that the surface-exposed adhesin promotes cellular invasion and placental colonization. Infect. Immun..

[96] Fardini Y., Wang X., Témoin S., Nithianantham S., Lee D., Shoham M., Han Y.W. (2011). *Fusobacterium nucleatum* adhesin FadA binds vascular endothelial cadherin and alters endothelial integrity. Mol. Microbiol..

[97] Abed J., Emgård J.E., Zamir G., Faroja M., Almogy G., Grenov A., Sol A., Naor R., Pikarsky E., Atlan K.A. (2016). Fap2 mediates *Fusobacterium nucleatum* colorectal adenocarcinoma enrichment by binding to tumor-expressed Gal-GalNAc. Cell Host Microbe.

[98] Parhi L., Abed J., Shhadeh A., Alon-Maimon T., Udi S., Ben-Arye S.L., Tam J., Parnas O., Padler-Karavani V., Goldman-Wohl D. (2022). Placental colonization by *Fusobacterium nucleatum* is mediated by binding of the Fap2 lectin to placentally displayed Gal-GalNAc. Cell Rep..

[99] Parhi L., Alon-Maimon T., Sol A., Nejman D., Shhadeh A., Fainsod-Levi T., Yajuk O., Isaacson B., Abed J., Maalouf N. (2020). Breast cancer colonization by *Fusobacterium nucleatum* accelerates tumor growth and metastatic progression. Nat. Commun..

[100] Gur C., Ibrahim Y., Isaacson B., Yamin R., Abed J., Gamliel M., Enk J., Bar-On Y., Stanietsky-Kaynan N., Coppenhagen-Glazer S. (2015). Binding of the Fap2 protein of *Fusobacterium nucleatum* to human inhibitory receptor TIGIT protects tumors from immune cell attack. Immunity.

[101] Kolenbrander P.E., Andersen R.N., Blehert D.S., Egland P.G., Foster J.S., Palmer R.J. (2002). Communication among oral bacteria. Microbiol. Mol. Biol. Rev..

[102] Perry M.B., MacLean L.M., Brisson J.R., Wilson M.E. (1996). Structures of the antigenic O-polysaccharides of lipopolysaccharides produced by *Actinobacillus actinomycetemcomitans* serotypes a, c, d and e. Eur. J. Biochem..

[103] Kaplan J.B., Perry M.B., MacLean L.L., Furgang D., Wilson M.E., Fine D.H. (2001). Structural and genetic analyses of O polysaccharide from *Actinobacillus actinomycetemcomitans* serotype f. Infect. Immun..

[104] Takada K., Saito M., Tsuzukibashi O., Kawashima Y., Ishida S., Hirasawa M. (2010). Characterization of a new serotype g isolate of *Aggregatibacter actinomycetemcomitans*. Mol. Oral Microbiol..

[105] Perry M.B., MacLean L.L., Gmür R., Wilson M.E. (1996). Characterization of the O-polysaccharide structure of lipopolysaccharide from *Actinobacillus actinomycetemcomitans* serotype b. Infect. Immun..

[106] Karched M., Bhardwaj R.G., Asikainen S.E. (2015). Coaggregation and biofilm growth of *Granulicatella* spp. with *Fusobacterium nucleatum* and *Aggregatibacter actinomycetemcomitans*. BMC Microbiol..

[107] Rupani D., Izano E.A., Schreiner H.C., Fine D.H., Kaplan J.B. (2008). *Aggregatibacter actinomycetemcomitans* serotype f O-polysaccharide mediates coaggregation with *Fusobacterium nucleatum*. Oral Microbiol. Immunol..

[108] Tanaka Y., Oogai Y., Matsumoto A., Noguchi K., Nakata M. (2025). The outer membrane autotransporters Fap2 and CmpA facilitate specific coaggregation between *Fusobacterium nucleatum* and *Aggregatibacter actinomycetemcomitans* serotypes b and d. Appl. Environ. Microbiol..

[109] Wu C., Chen Y.W., Scheible M., Chang C., Wittchen M., Lee J.H., Luong T.T., Tiner B.L., Tauch A., Das A. (2021). Genetic and molecular determinants of polymicrobial interactions in *Fusobacterium nucleatum*. Proc. Natl. Acad. Sci. USA.

[110] Kolenbrander P.E., Andersen R.N. (1989). Inhibition of coaggregation between *Fusobacterium nucleatum* and *Porphyromonas (Bacteroides) gingivalis* by lactose and related sugars. Infect. Immun..

[111] Rosen G., Sela M.N. (2006). Coaggregation of *Porphyromonas gingivalis* and *Fusobacterium nucleatum* PK 1594 is mediated by capsular polysaccharide and lipopolysaccharide. FEMS Microbiol. Lett..

[112] Coppenhagen-Glazer S., Sol A., Abed J., Naor R., Zhang X., Han Y.W., Bachrach G. (2015). Fap2 of *Fusobacterium nucleatum* is a galactose-inhibitable adhesin involved in coaggregation, cell adhesion, and preterm birth. Infect. Immun..

[113] Liu P.F., Shi W., Zhu W., Smith J.W., Hsieh S.L., Gallo R.L., Huang C.M. (2010). Vaccination targeting surface FomA of *Fusobacterium nucleatum* against bacterial co-aggregation: Implication for treatment of periodontal infection and halitosis. Vaccine.

[114] Okuda T., Kokubu E., Kawana T., Saito A., Okuda K., Ishihara K. (2012). Synergy in biofilm formation between *Fusobacterium nucleatum* and *Prevotella* species. Anaerobe.

[115] Sharma A., Inagaki S., Sigurdson W., Kuramitsu H.K. (2005). Synergy between *Tannerella forsythia* and *Fusobacterium nucleatum* in biofilm formation. Oral Microbiol. Immunol..

[116] Posch G., Pabst M., Brecker L., Altmann F., Messner P., Schäffer C. (2011). Characterization and scope of S-layer protein O-glycosylation in *Tannerella forsythia*. J. Biol. Chem..

[117] Rosen G., Genzler T., Sela M.N. (2008). Coaggregation of *Treponema denticola* with *Porphyromonas gingivalis* and *Fusobacterium nucleatum* is mediated by the major outer sheath protein of *Treponema denticola*. FEMS Microbiol. Lett..

[118] Kaplan C.W., Lux R., Haake S.K., Shi W. (2009). The *Fusobacterium nucleatum* outer membrane protein RadD is an arginine-inhibitable adhesin required for inter-species adherence and the structured architecture of multispecies biofilm. Mol. Microbiol..

[119] Bibek G.C., Wu C. (2025). The CarSR two-component system directly controls *radD* expression as a global regulator that senses bacterial coaggregation in *Fusobacterium nucleatum*. J. Bacteriol..

[120] Lima B.P., Shi W., Lux R. (2017). Identification and characterization of a novel *Fusobacterium nucleatum* adhesin involved in physical interaction and biofilm formation with *Streptococcus gordonii*. Microbiologyopen.

[121] Hashimoto M., Ogawa S., Asai Y., Takai Y., Ogawa T. (2003). Binding of *Porphyromonas gingivalis* fimbriae to *Treponema denticola* dentilisin. FEMS Microbiol. Lett..

[122] Yamada M., Ikegami A., Kuramitsu H.K. (2005). Synergistic biofilm formation by *Treponema denticola* and *Porphyromonas gingivalis*. FEMS Microbiol. Lett..

[123] Yoshikawa K., Kikuchi Y., Kokubu E., Imamura K., Saito A., Ishihara K. (2018). Identification of a specific domain of *Porphyromonas gingivalis* Hgp44 responsible for adhesion to *Treponema denticola*. Pathog. Dis..

[124] Jung Y.J., Jun H.K., Choi B.K. (2016). Gingipain-dependent augmentation by *Porphyromonas gingivalis* of phagocytosis of *Tannerella forsythia*. Mol. Oral Microbiol..

[125] Śmiga M., Olczak T. (2019). PgRsp is a novel redox-sensing transcription regulator essential for *Porphyromonas gingivalis* virulence. Microorganisms.

[126] Ikegami A., Honma K., Sharma A., Kuramitsu H.K. (2004). Multiple functions of the leucine-rich repeat protein LrrA of *Treponema denticola*. Infect. Immun..

[127] Sano Y., Okamoto-Shibayama K., Tanaka K., Ito R., Shintani S., Yakushiji M., Ishihara K. (2014). Dentilisin involvement in coaggregation between *Treponema denticola* and *Tannerella forsythia*. Anaerobe.

[128] Lima B.P., Hu L.I., Vreeman G.W., Weibel D.B., Lux R. (2019). The Oral Bacterium *Fusobacterium nucleatum* binds *Staphylococcus aureus* and alters expression of the staphylococcal accessory regulator *sarA*. Microb. Ecol..

[129] Andersen R.N., Ganeshkumar N., Kolenbrander P.E. (1998). *Helicobacter pylori* adheres selectively to *Fusobacterium* spp. *Oral Microbiol*. Immunol..

[130] Engevik M.A., Danhof H.A., Auchtung J., Endres B.T., Ruan W., Bassères E., Engevik A.C., Wu Q., Nicholson M., Luna R.A. (2021). *Fusobacterium nucleatum* adheres to *Clostridioides difficile* via the RadD adhesin to enhance biofilm formation in intestinal mucus. Gastroenterology.

[131] Yang L., Sriram G., Chew R.J.J., Tan K.S. (2025). *Limosilactobacillus reuteri*-*Fusobacterium nucleatum* interactions modulate biofilm composition and immunogenicity. J. Periodontal Res..

